# Effectiveness of a Web-Based Physical Activity Intervention in Patients With Knee and/or Hip Osteoarthritis: Randomized Controlled Trial

**DOI:** 10.2196/jmir.2662

**Published:** 2013-11-22

**Authors:** Daniël Bossen, Cindy Veenhof, Karin EC Van Beek, Peter MM Spreeuwenberg, Joost Dekker, Dinny H De Bakker

**Affiliations:** ^1^Netherlands Institute for Health Services Research (NIVEL)UtrechtNetherlands; ^2^EMGO InstituteDepartment of Rehabilitation Medicine & Department of PsychiatryVU University Medical Center AmsterdamAmsterdamNetherlands; ^3^TranzoTilburg UniversityTilburgNetherlands

**Keywords:** physical activity, osteoarthritis, Web-based intervention, randomized controlled trial

## Abstract

**Background:**

Patients with knee and/or hip osteoarthritis (OA) are less physically active than the general population, while the benefits of physical activity (PA) have been well documented. Based on the behavioral graded activity treatment, we developed a Web-based intervention to improve PA levels in patients with knee and/or hip OA, entitled “Join2move”. The Join2move intervention is a self-paced 9-week PA program in which the patient’s favorite recreational activity is gradually increased in a time-contingent way.

**Objective:**

The aim of the study was to investigate whether a fully automated Web-based PA intervention in patients with knee and/or hip OA would result in improved levels of PA, physical function, and self-perceived effect compared with a waiting list control group.

**Methods:**

The study design was a two-armed randomized controlled trial which was not blinded. Volunteers were recruited via articles in newspapers and health-related websites. Eligibility criteria for participants were: (1) aged 50-75 years, (2) self-reported knee and/or hip OA, (3) self-reported inactivity (30 minutes of moderate PA, 5 times or less per week), (4) no face-to-face consultation with a health care provider other than general practitioners, for OA in the last 6 months, (5) ability to access the Internet weekly, and (6) no contra-indications to exercise without supervision. Baseline, 3-month, and 12-month follow-up data were collected through online questionnaires. Primary outcomes were PA, physical function, and self-perceived effect. In a subgroup of participants, PA was measured objectively using accelerometers. Secondary outcomes were pain, fatigue, anxiety, depression, symptoms, quality of life, self-efficacy, pain coping, and locus of control.

**Results:**

Of the 581 interested respondents, 199 eligible participants were randomly assigned to the intervention (n=100) or waiting list control group (n=99). Response rates of questionnaires were 84.4% (168/199) after 3 months and 75.4% (150/199) after 12 months. In this study, 94.0% (94/100) of participants actually started the program, and 46.0% (46/100) reached the adherence threshold of 6 out of 9 modules completed. At 3 months, participants in the intervention group reported a significantly improved physical function status (difference=6.5 points, 95% CI 1.8-11.2) and a positive self-perceived effect (OR 10.7, 95% CI 4.3-26.4) compared with the control group. No effect was found for self-reported PA. After 12 months, the intervention group showed higher levels of subjective (difference=21.2 points, 95% CI 3.6-38.9) and objective PA (difference=24 minutes, 95% CI 0.5-46.8) compared with the control group. After 12 months, no effect was found for physical function (difference=5 points, 95% CI −1.0 to 11.0) and self-perceived effect (OR 1.2, 95% CI 0.6-2.4). For several secondary endpoints, the intervention group demonstrated improvements in favor of the intervention group.

**Conclusions:**

Join2move resulted in changes in the desired direction for several primary and secondary outcomes. Given the benefits and its self-help format, Join2move could be a component in the effort to enhance PA in sedentary patients with knee and/or hip OA.

**Trial Registration:**

The Netherlands National Trial Register: NTR2483; http://www.trialregister.nl/trialreg/admin/rctview.asp?TC=2483 (Archived by WebCite at http://www.webcitation.org/67NqS6Beq).

## Introduction

It has been recognized that regular physical activity (PA) positively impacts the severity and course of numerous chronic diseases [1,2]. Among patients with knee and/or hip osteoarthritis (OA), regular PA has proven to be beneficial in preserving physical function and reducing pain symptoms [3,4]. Improvement in physical function and reduction in pain are positively related to several psychological factors and thus may affect self-esteem, pain coping, and self-efficacy in patients with knee and/or hip OA [5,6]. However, due to pain and other symptoms, patients with OA are less physically active than the general population [7,8]. Therefore, PA as a nonpharmacological intervention has been advocated in the treatment of OA patients [9].

Since OA is mainly managed within primary care, general practitioners (GPs) are advised to stimulate patients to adopt and maintain higher levels of PA. In practice, however, a GP’s ability to encourage physical exercise is limited by time constraints and lack of standard protocols [10-12]. At the same time, it is unlikely that patients with knee and/or hip OA receive help elsewhere, since patients are not referred to other health care professionals [13] and because people often view their peripheral joint pain as an inevitable part of aging [14]. Numerous patients lack knowledge and skills to modify their PA routines and have negative concerns (eg, fear of pain and catastrophizing thoughts) about the impact of PA on their joints [15,16].

In an attempt to promote a more physically active lifestyle among patients with knee and/or hip OA, effective PA interventions are needed. With the explosion of Internet accessibility, Web-based interventions seem to provide a novel medium to reach patients with knee and/or hip OA; 61% of Europeans and 79% of North Americans have Internet access [17]. In the Netherlands, 95% of adults (55-65 years) and 75% of older adults (65-75 years) have access to Internet in their home [18]. Web-based interventions are applications available through a website with the intent to enhance understanding of a health condition and to change health behavior. In particular, Web-based interventions with minimal human contact have the potential of high reach, low costs, and are accessible anytime and anywhere [19]. Previous Web-based interventions for inactive populations and patients with a chronic disease (eg, diabetes, cardiovascular diseases, and chronic obstructive pulmonary disease) have produced inconclusive findings [20-22].

To date, there are no Web-based PA interventions for patients with knee and/or hip OA that we know of. Given the advantages of the Internet, we developed “Join2move”. The Join2move program differs from existing Web-based programs since it focuses on knee and hip OA and strategies to enhance PA despite the presence of pain. The design is inspired by a previously developed exercise program known as the behavior graded activity (BGA) program [23]. The BGA treatment is an exercise regimen based on operant behavior principles that stimulate OA patients to gradually increase their daily life activities for fixed time periods. In accordance with the BGA treatment, Join2move intervention is a 9-week PA program in which the patient’s favorite recreational activity is gradually increased in a time-contingent way. The intensity of the modules is predetermined by the participants themselves. To investigate the effectiveness of Join2move, we compared the Web-based intervention versus no intervention. This study aimed to answer the following research question: “What is the short (3 months) and long-term (12 months) effectiveness of the Join2move intervention in patients with knee and/or hip OA in PA, physical function, and self-perceived effect in comparison with a waiting list control group?”

## Methods

### Study Design

This study was a two-armed, 12-month, randomized controlled trial (RCT) with continuous recruitment and data collection. Allocation ratio was 1:1 and enrollment started on January 3, 2011, and ended November 5, 2011. The trial is reported according to the CONSORT-EHEALTH checklist [24]. Ethics approval was obtained from the medical ethics committee of the VU University Medical Center, Amsterdam.

### Participants

Patients with self-reported knee and/or hip OA were recruited through advertisements in Dutch newspapers and online on health-related websites. The advertisements briefly explained the purpose of the project and the beneficial health effects of PA. Interested individuals were referred to an open access study website and invited to complete an online eligibility questionnaire. Participants’ email addresses were used to contact them for online follow-up questionnaires, and home addresses were used for sending an information letter, informed consent form, and accelerometer. The eligibility criteria for participants were: (1) aged 50-75 years, (2) self-reported OA in knee and/or hip, (3) self-reported inactivity (<30 minutes of moderate PA three or five times or less per week), (4) no face-to-face consultation for OA with a health care provider, other than GP, in the last 6 months, (5) ability to access the Internet weekly, and (6) no contra-indications to exercise without supervision. Self-reported OA was determined by asking participants if they had a painful knee or hip joint and if a doctor or other health care provider had ever told them this was a result of OA. Contra-indication was determined by the PA-readiness questionnaire (PARQ) [25]. The PARQ questionnaire is designed to identify persons for whom increased PA may be contra-indicated. If patients filled out “no” to all questions, it was considered safe for the patients to engage Join2move. If participants answered “yes” to any of the seven PARQ questions, they were advised to see their GP before participation. Written medical clearance from a GP was not required.

### Procedure

Interested patients who met the inclusion criteria were sent an invitation letter with informed consent. Once informed consent was obtained, participants were invited to fill out an online baseline questionnaire. When baseline assessments were completed, participants were randomly assigned to the intervention (n=100) or control group (n=99). For concealment, a researcher (CV), not involved in data collection, distributed sequentially numbered opaque sealed envelopes with allocation details. Each sealed envelope was opened after the participant had given their written consent to participate in the study. After randomization, all participants were informed through email of their group assignment. Participants in the intervention group received a username and password to log in. Due to the nature of the study (waiting list controlled), neither the study staff nor the participants were blinded to group allocation. To assess the effectiveness of the Join2move intervention, we conducted two post measurements at 3 months and 12 months. At these follow-up times, all participants received online questionnaires. In addition to the online questionnaires, a random subgroup from both groups (n=83) received and returned an accelerometer by post. The decision for sending accelerometers to a subgroup of participants was made based on time and cost savings. An email and telephone reminder was used when participants failed to complete their online questionnaire within 2 weeks. Apart from sending accelerometers and telephone reminders, the study used an automated design. There was no face-to-face contact with study subjects.

### Development of the Intervention

Over the course of 1 year, a team of experts from the Netherlands institute for health services research (NIVEL) developed the program. During the development phase, an iterative design methodology [26] was used to test, analyze, and refine the Join2move intervention. We conducted a focus group (n=5), in home observations (n=4), a pilot study (n=20), and interviews (n=16). Furthermore, two usability methods (heuristic evaluation and a thinking aloud approach) were applied to determine the usability of the Web-based program. End-users (ie, patients with knee and/or hip OA) were involved continuously throughout the development process. The final version was used for the RCT study. No content changes were made during the trial period. Further details about the development are described elsewhere [27]. Participants involved in the focus group, pilot, and usability studies did not participate in the RCT study.

### The Intervention

**Figure 1:**
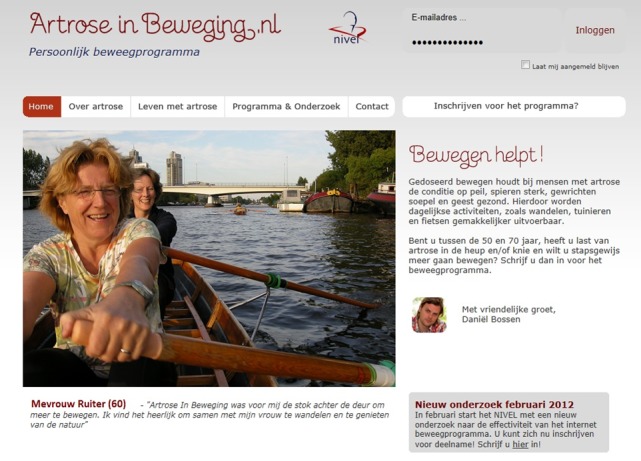
Join2move homepage.

The Join2move intervention is based on a previously developed and evaluated BGA program for patients with knee and/or hip OA [23]. The BGA program incorporates a baseline test, goal setting, time-contingent PA objectives (ie, on fixed time points), and text messages to promote PA. An essential feature of the BGA program is the positive reinforcement of gradual PA, despite the presence of pain. The gradual increase in activities changes the perception that PA is related to pain and reinforces confidence to improve PA performance [28]. The Join2move intervention is a fully automated Web-based intervention that contains automatic functions (web-based text messaging and automatic emails) without human support. Screenshots illustrating different stages of the Join2move intervention are presented in Multimedia Appendix 1. Participants are initially presented with a homepage (see Figure 1). The password-secured PA program is available 24/7 from the homepage and is provided without charge. In keeping with the BGA treatment, the Join2move intervention is a self-paced 9-week PA program in which a patient’s favorite recreational activity is gradually increased in a time-contingent way. In the first week of the program, users select a central activity such as cycling, walking, or gardening; perform a 3-day self-test; and determine a short-term goal for the next 8 weeks. Based on test performances and a short-term goal, 8 tailored weekly modules are automatically generated. Every week, new modules are posted on the password-secured website. Modules remain on the website for 1 week. After 7 days, users are presented with an evaluation form about pain and performance. Pain is assessed with a 10-point Numerical Rating Scale (0 is no pain, 10 is worst possible pain). Performance was measured by three items, namely: (1)“I completed the module as instructed”, (2) “I did more than the instructed module”, or (3) “I did less than the instructed module” due to “(a) time constraints, (b) weather conditions, (c) pain in my knee and/or hip, and (d) other physical complaints”. Subsequently, tailored to the answers from the evaluation form, automated text-based messages were generated. Furthermore, if users indicated that a module was missed due to time constraints or weather conditions, they had the option to repeat the current module or to continue with the next module. If users indicated that a module was missed due to pain in knee/ hip or other physical complaints, they had the ability to repeat the module (a maximum of three times), adapt the intensity of the module, or proceed with the next module. In addition to the weekly modules, information about OA, lifestyle, and videos are provided. Since personal messages are updated on a weekly basis, users are encouraged to log in once a week. Automatic emails are generated if participants do not log on to the website for two weeks. At the end of the program, the website presents a motivational message to perform regular PA in the future.

### Waiting List

In this study, we used a waiting list control group. The control group (as well as the intervention group) received a letter with information about the study, PA, and OA. During the follow-up period, participants from the control group had no contact with participants from the intervention group and no access to the Join2move intervention. After the follow-up period, patients in the waiting list group received access to the Join2move intervention.

### Measures

Three online questionnaires (0, 3, and 12 months) were used for data collection and a subgroup of participants received an accelerometer to measure PA. Questionnaires were created by online survey experts from the NIVEL institute and tested among a pilot study of 20 participants prior to the RCT study [27]. All participants received an email with a URL link to an online questionnaire. We offered no incentives to complete questionnaires.

### Demographic and Clinical Outcomes

Gender, education (low: primary and lower vocational education; middle: secondary and middle vocational education; high: higher vocational and university education), body height (centimeters), age (years), body weight (kilograms), location of OA complaints (knee, hip, or both), duration of OA complaints (years and months), and presence of comorbid conditions were obtained. Body mass index (BMI) was calculated as the weight in kilograms, divided by the height in meters squared.

### Program Usage

Program usage was measured by the number of weekly modules completed. A module consisted of a text-based assignment and accompanying evaluation form, which was presented on the website for 7 consecutive days. Once a participant read the weekly assignments and filled out the evaluation form, the module was defined as completed and the user was automatically presented with a new module. In total, there were nine weekly modules that could have been opened by the participant. This was automatically registered. Adequate program use was defined if users completed at least 6 out of 9 modules. Intervention supplements (ie, videos and general information on the homepage) were not included in the adherence measure.

### Primary Outcome Measures

#### Physical Activity

Self-reported PA was measured by the validated PA Scale for the Elderly (PASE) [29]. The PASE questionnaire is designed to assess PA patterns in older adults. The instrument consists of questions on household, leisure time, and work-related activities. The activities (assigned according to the level of intensity: light, moderate, and strenuous) are recorded as never, seldom (1-2 days/week), sometimes (3-4 days/week), or often (5-7 days/week). The amount of time spent in each activity is multiplied by its intensity. In addition to the PASE questionnaire, assessment of PA was supported through ActiGraph GT3X tri-axial accelerometers [30]. A random subsample of participants from the intervention and control groups were invited to wear this accelerometer. In total, 83 accelerometers were distributed by post to 41 controls and 42 participants in the intervention group. Participants were instructed to wear the monitor on a belt around their waist for 5 consecutive days [31], except during sleeping, showering, or swimming. In addition, participants were requested to fill out a short activity diary. This diary contained questions about wearing time, unusual activities, and reasons for device removal. When accelerometers and diaries were returned by post, data were downloaded, processed, and subsequently analyzed. Participants with at least 10 hours of PA data for at least 4 valid days were included for further analysis. In order to determine the actual PA thresholds, the widely accepted thresholds by Freedson et al [32] were used: 0-99 counts for sedentary activities, 100-1951 for light PA, 1952-5724 moderate PA, 5725-9498 for vigorous PA, and 9499-max for very vigorous activities. The total time spent in light, moderate, and (very) vigorous PA was summed and subsequently divided by the number of days worn to compute the daily average time spent in total activity. For analysis, data were recorded at 1-minute intervals. Sequences of at least 60 minutes of zero counts were defined as non-wearing time. Although the accelerometer was tri-axial, only the vertical axis was used for analysis. This was decided since preprogrammed thresholds of the tri-axial model have yet to be determined [33].

#### Physical Function

Physical function was determined by a subscale of the Knee OA Outcome Score (KOOS) [34,35] and the Hip Injury OA Outcome Score (HOOS) [36,37]. The KOOS and HOOS are self-administered questionnaires to assess patients’ opinions about their knee and/or hip-related problems according to five indicators on a 5-point Likert scale: (1) pain, (2) symptoms, (3) physical function, (4) sport and recreation function, and (5) quality of life.

#### Self-Perceived Effect

At 3 months and 12 months, self-perceived effect was assessed by a single question that asked participants about the degree of change since their previous assessment. We used a 7-point Likert scale ranging from “much worse” to “much better”, with “about the same” located in the middle. The outcomes of self-perceived effect were dichotomized into “improved” (much better, better, and slightly better) and “not improved” (about the same, slightly worse, worse, much worse).

### Secondary Outcomes

Pain and fatigue were assessed with a 10-point Numerical Rating Scale (0 is no pain/not tired and 10 is worst possible pain/very tired). OA-related symptoms, quality of life, and sport and recreation were measured with a subscale of the HOOS and KOOS. Anxiety and depression were evaluated by the 14-item Hospital Anxiety and Depression Scale (HADS) [38]. Self-efficacy for pain and other symptoms was evaluated by using the Arthritis Self-Efficacy Scale [39,40]. Active and passive pain coping were determined by the Pain Coping Inventory questionnaire [41]. Locus of control (people’s belief that health is or is not determined by their behavior) was examined with the Multidimensional Health Locus of Control Scale [42].

### Sample Size

Sample size calculations were performed. Since no previous research has provided adequate statistical information on PA, power calculations were based on physical function and self-perceived effect. We needed 200 patients with knee and/or hip OA in total to detect a small to medium effect (0.2-0.5) in the outcome measure physical functioning and self-perceived effect (25% difference). Conventional levels of statistical power (0.8) and level of statistical significance (*P*=.05) were used.

### Statistical Analysis

Findings were analyzed using an intention-to-treat analysis. Complementary to the primary analysis, per-protocol analysis was employed using only adherent patients in the intervention group (at least 6 out of 9 modules completed) and the entire control group. A nonresponse analysis was carried out in order to examine differences among participants who completed the questionnaires and participants who did not. Furthermore, we compared primary baseline variables between the response and the nonresponse group in order to investigate selective attrition. A Generalized Estimating Equations (GEE) approach controlling for baseline values, age, OA location, and gender was used to analyze effects of the intervention on primary and secondary outcomes. An independent correlation structure was used to account for the within-subject correlations. Also, *t* tests and chi-square tests were used to compare baseline characteristics in the intervention and control group to perform nonresponse analysis and to determine selective attrition. Between-group effect sizes (ES) were calculated according to Cohen’s *d*. Traditionally, ES of ≥0.8 are interpreted as “large” effects, effect sizes of 0.5 as “moderate”, and effect sizes of ≤0.2 as “small” effects [43]. The effect size for self-perceived effect was given by odds ratios (OR). Since GEE analyses are tolerant to data missing, no imputation techniques were used [44].

## Results

### Participant Characteristics and Study Participation

**Figure 2:**
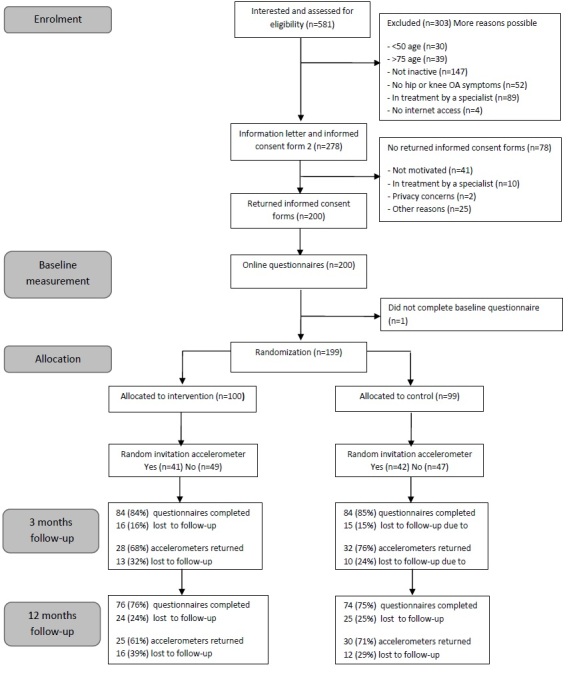
Flow of participants throughout the trial.

Figure 2 depicts the flow of participants throughout the trial. In total, 581 persons were screened, 278 (47.8%, 278/581) were eligible, and 200 (71.9%, 200/581) consented to participate. Finally, a total of 99 participants were assigned to the control group, and 100 participants were allocated to the experimental group. With regard to the questionnaires, the overall response rate was 84.4% (168/199) after 3 months and 75.4% (150/199) after 12 months. With respect to the subgroup of participants who wore an accelerometer (n=83), the overall response rate was 72% (60/83) and 66% (55/83) after 12 months. Reasons for not participating in the follow-up surveys were health/medical issues (37%, 17/46), lack of motivation (15%, 7/46), personal/family reasons (13%, 6/46), other (13%, 6/46), and unknown reasons (22%, 10/46).

Table 1 presents participants’ characteristics and primary outcome measures at baseline. Participants were predominantly female (64.8%, 129/199), had knee OA (63.8%, 127/199), and no comorbidity (62.8%, 125/199). Mean age was 62 years (SD 5.7) and mean BMI was 27.6 (SD 4.5). Of the participants, 45.7% (91/199) had a high level of education and 9.0% (18/199) had OA symptoms for less than 1 year. Demographic baseline values were not statistically different between the two groups. Those who did not complete follow-up questionnaires were more likely to have at least one comorbidity (*P*=.01) than those who did. With respect to other baseline characteristics, no differences were found (data not shown). The subgroup of participants (n=83) who wore an accelerometer did not differ from the other participants (n=116) on baseline characteristics (data not shown).

**Table 1:** Baseline demographic and clinical characteristics.

Characteristic		Intervention, n=100	Control, n=99	*P* value
**Gender, n (%)**				
	Male	40 (40.0)	30 (30.3)	.15
	Female	60 (60.0)	69 (69.7)	
Age (years), mean (SD)		61 (5.9)	63 (5.4)	.05
BMI (kg/m^2^), mean (SD)		27.6 (4.6)	27.5 (4.5)	.79
**Location OA, n (%)**				
	Knee	67 (67.0)	60 (60.6)	.37
	Hip	21 (21.0)	20 (20.2)	
	Both	12 (12.0)	19 (19.2)	
**Duration of symptoms, n (%)**				
	≤1 year	12 (12.0)	6 (6.1)	.33
	>1-3 years	28 (28.0)	27 (27.3)	
	>3-7 years	27 (27.0)	27 (27.3)	
	≥7 years	33 (33.0)	39 (39.4)	
**Education**				
	Low education	13 (13.0)	15 (15.2)	.36
	Middle education	36 (36.0)	43 (43.4)	
	High education	51 (51.0)	40 (40.4)	
**Comorbidity, n (%)**				
	None	65 (65.0)	60 (60.6)	.43
	One	19 (19.0)	16 (16.2)	
	Two or more	16 (16.0)	23 (23.2)	

### Program Usage

**Figure 3:**
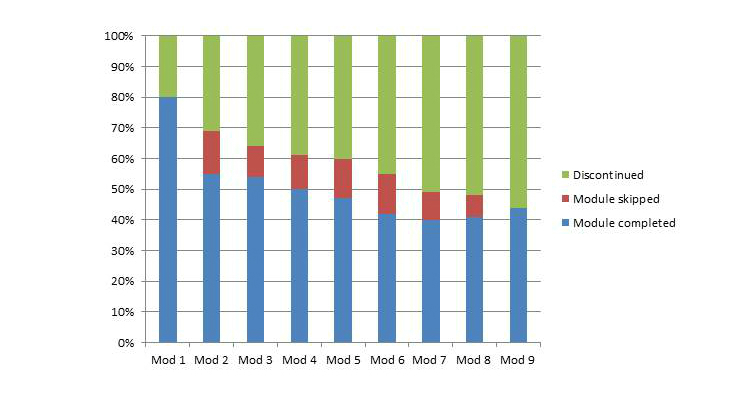
Module completion rate.

Of the 100 participants who received a password and username to enroll, 94.0% (94/100) made a start with the first module and 6.0% (6/100) never logged in to their personal website. Figure 3 depicts an overview of the module completion rate. The first module was completed by 80.0% (80/100) of subjects. This ratio declined to 55.0% (55/100) during the second module. This percentage of completed modules remained steady up to the end of the program. Of the 94 participants who started the program, the average completion was 5.6 (SD 2.9) out of nine modules. Participants selected walking (46.0%, 46/100), cycling (32.0%, 32/100), Nordic walking (4.0%, 4/100), gardening (4.0%, 4/100), and other activities (8.0%, 8/100) as central activity; 6.0% (6/100) of the potential users never selected a central activity because they never logged in to their website. Since personal messages were updated on a weekly basis, patients had the opportunity to complete a module within 7 days. When a module was missed, users still had the ability to complete the next module. Finally, 19.0% (19/100) of the participants fulfilled all modules of the program, and 46.0% (46/100) reached the threshold of adherence (executed at least 6 out of 9 modules). The presence of comorbidity seemed a predictor for nonusage. Of the patients with an additional disease, 71% (25/35) were nonadherent to the Join2move intervention. This percentage was substantially lower among those without a comorbidity, namely 45% (29/65). Adverse events, such as extreme pain and injuries, were not reported during the intervention period.

### Primary Outcome Measures

Table 2 presents results of the primary outcome measures at 3 and 12 months. At 3 months, participants in the intervention group reported a significantly improved physical function status (*P*=.006, *d*=0.20) and a positive self-perceived effect (*P*<.001; OR 10.7, 95% CI 4.3-26.4). No effect was found for PA measured with the PASE questionnaire (*P*=.84, *d*=−0.01) and accelerometer (*P*=.83, *d*=0.02). After 12 months, the intervention group showed higher levels of subjective and objective PA (*P*=.02, *d*=0.18 and *P*=.045, *d*=0.19) compared with the control group. At 12 months, no effect was found for physical function (*P*=.10, *d*=0.17) and self-perceived effect (*P*=.50; OR 1.2, 95% CI 0.6-2.4). The accelerometer group (n=83) did not differ from the group who did not wear an accelerometer (n=118) with respect to short and long-term PASE scores (data not shown).

### Secondary Outcome Measures

Table 3 presents results of the secondary outcome measures at 3 months and 12 months. At 3 months, we observed statistically significant differences between the intervention and control group with respect to pain (*P*=.002; *d*=−0.2), tiredness (*P*=.04, *d*=−0.16), and improvements in self-efficacy for pain (*P*=.008, *d*=0.17) in favor of the intervention group. Other secondary endpoints were not significantly different between the two groups. At 12 months, subjects in the intervention group reported less tiredness (*P*=.008; *d*=−0.22), better passive pain coping scores (*P*=.008, *d*=−0.18), and reduced anxiety levels (*P*=.007; *d*=−0.21) compared to those in the control group. Other secondary outcomes were not significantly different between the conditions at 12 months.

### Per-Protocol Analyses

The per-protocol analysis—a comparison of the adherent patients in the intervention group (ie, participants who completed 6 out of 9 week modules) and the entire control group—yielded positive self-perceived effects in favor of the intervention group (data not presented). Higher levels of participation had no influence on other primary and secondary outcomes (data not presented).

**Table 2:** Primary outcome measures: improvements and differences between groups^a^.

Outcome measures		n	Intervention, mean (95% CI)	n	Control, mean (95% CI)	Difference, I-C^b^(95% CI)	ES	*P* value
**Total PA, PASE (0-400)**								
	Baseline	100	163 (130-196)	97	160 (123-197)	—	—	—
	3 months	85	162 (136-187)	79	163 (137-190)	−1.6 (−16.6 to 13.5)	−0.01	.84
	12 months	74	174 (150-198)	71	153 (125-181)	21.2 (3.6-38.9)	0.18	.02
**Total PA (accelerometer min/day)**								
	Baseline	39	369 (299-439)	40	395 (322-468)	—	—	—
	3 months	27	361 (312-411)	30	358 (310-407)	3 (−26 to 32)	0.02	.83
	12 months	24	361 (317-406)	28	338 (291-384)	24 (0.5-46.8)	0.19	.045
**Physical functioning (0-100)**								
	Baseline	99	58.8 (51.5-66.0)	98	55.2 (47.9-62.5)	—	—	—
	3 months	84	67.8 (59.2-76.4)	80	61.3 (52.7-69.9)	6.5 (1.8-11.2)	0.20	.006
	12 months	75	67.9 (59.1-76.7)	72	62.9 (54.1-71.7)	5.0 (−1.0 to 11.0)	0.17	.1
**Self-perceived effect (improved-not improved)**								
	3 months, n (%) improved	85	44 (44)	83	7 (7.1)	10.7^c^ (4.3-26.4)	—	<.001
	12 months, n (%) improved	76	34 (34)	74	27 (27.3)	1.2^c^ (0.6-2.4)	—	.5

^a^For PA, physical functioning, and self-perceived effect, a higher score indicates an improvement. Results are based on GEE analyses and adjusted for corresponding baseline variables, age, OA location, and gender.

^b^I-C: difference between intervention and control group.

^c^odds ratio

**Table 3:** Secondary outcome measures: improvements and differences between groups^a^.

Outcome measures		n	Intervention, mean (95% CI)	n	Control, mean (95% CI)	Difference, I-C^b^(95% CI)	ES	*P* value

**Sedentary intensity (accelerometer min/day)**								
	Baseline	39	571 (498-645)	40	555 (479-630)	—	—	—
	3 months	27	508 (454-563)	30	540 (477-603)	−32 (−67.7 to 3.7)	−0.20	.08
	12 months	24	514 (448-580)	28	531 (467-595)	−17 (−54.7 to 20.7)	−0.10	.38
**Pain (0-10)**								
	Baseline	100	5.4 (4.2-6.5)	98	4.9 (3.7-6.1)			
	3 months	85	3.5 (2.5-4.6)	81	4.5 (3.4-5.7)	−1 (−1.6 to −0.38)	−0.20	.002
	12 months	76	3.5 (2.4-4.5)	71	3.8 (2.7-4.9)	−0.36 (−1.1 to 0.38)	−0.07	.33
**Tiredness (0-10)**								
	Baseline	100	5.6 (4.3-6.9)	99	5.5 (4.3-6.8)	—	—	—
	3 months	85	3.2 (2-4.4)	81	4.1 (2.9-5.3)	−0.84 (−1.6 to -0.06)	−0.16	.04
	12 months	76	3 (1.9-4.2)	71	4.1 (3-5.2)	−1.15 (−1.9 to −0.28)	−0.22	.008
**Symptoms (0-100)**								
	Baseline	100	68.2 (60.2-76.2)	99	70.9 (62.7-79.2)	—	—	—
	3 months	85	67.4 (59.1-75.8)	80	64.3 (55.3-73.2)	3.1 (−1.3 to 7.6)	0.08	.16
	12 months	76	65.7 (57.4-74.0)	71	62.8 (53.4-72.1)	3 (−2.1 to 8.1)	0.08	.25
**Quality of life (0-100)**								
	Baseline	100	38 (30.6-45.5)	98	40.9 (33.6-48.2)	—	—	—
	3 months	85	49.4 (41.7-57.0)	80	47.3 (39.4-55.1)	2.1 (−1.7 to 5.9)	0.06	.28
	12 months	75	48.7 (40.8-56.6)	71	47.5 (39.3-55.6)	1.2 (−4.4 to 6.8)	0.03	.68
**Sport/recreation (0-100)**								
	Baseline	88	27.6 (14.7-40.4)	78	27.6 (13.4-41.9)	—	—	—
	3 months	58	42.6 (29.6-55.6)	55	42.6 (29-56.2)	0 (−8.0 to 8.1)	0	1
	12 months	53	42.4 (28.1-56.8)	47	39.6 (25.6-53.5)	2.9 (−6.3 to 12.1)	0.08	.54
**Self-efficacy pain (1-5)**								
	Baseline	100	4.1 (3.6-4.6)	97	3.8 (3.6-4.2)	—	—	—
	3 months	85	4 (3.6-4.4)	79	3.7 (3.3-4.1)	0.31 (0.1-0.5)	0.17	.008
	12 months	75	4 (3.6-4.4)	72	3.9 (3.5-4.3)	0.12 (−01 to 0.4)	0.06	.35
**Self-efficacy other symptoms (1-5)**								
	Baseline	100	3.6 (3.1-4.1)	96	3.8 (3.4-4.3)	—	—	—
	3 months	85	4 (3.7-4.4)	79	3.8 (3.7-4.4)	0.21 (0-0.4)	0.12	.07
	12 months	75	4.1 (3.7-4.4)	72	3.8 (3.5-4.2)	0.23 (0-0.5)	0.20	.05
**Active pain coping (0-4)**								
	Baseline	100	2.2 (2.0-2.4)	96	2.2 (2-2.4)	—	—	—
	3 months	83	2 (1.9-2.2)	77	2 (1.8-2.2)	−0.02 (−0.1 to 0.1)	−0.02	.81
	12 months	73	2 (1.8-2.2)	70	2 (1.8-2.2)	0 (−0.1 to 0.1)	0	.98
**Passive pain coping (0-4)**								
	Baseline	100	1.8 (1.7-2.0)	96	1.8 (1.6-1.9)	—	—	—
	3 months	83	1.7 (1.6-1.8)	77	1.7 (1.6-1.9)	−0.04 (−0.1 to 0.04)	0	.29
	12 months	73	1.7 (1.5-1.8)	70	1.8 (1.7-1.9)	−0.12 (−0.2 to –0.03)	−0.18	.008
**Internal locus of control (6-36)**								
	Baseline	100	27.1 (25.1-29.2)	96	27.5 (25.2-29.8)	—	—	—
	3 months	84	23.9 (21.9-25.8)	79	23.4 (21.3-25.6)	0.45 (−0.6 to 1.5)	0.06	.41
	12 months	74	23.6 (21.7-25.6)	70	24 (21.7-26.2)	−0.3 (−1.5 to 0.9)	−0.05	.61
**Powerful others locus of control (6-36)**								
	Baseline	99	17.4 (14.8-20.0)	96	18.8 (15.8-21.8)	—	—	—
	3 months	83	16.5 (15.0-18.0)	79	16.1 (14.3-18.0)	0.37 (−0.8 to 1.5)	0.05	.53
	12 months	73	15.2 (13.6-6.9)	70	16 (14.1-17.9)	−0.74 (−2.0 to 0.6)	−0.1	.26
**Anxiety (0-21)**								
	Baseline	100	4 (2.5-5.6)	97	4.2 (2.6-5.9)	—	—	—
	3 months	85	3.5 (2.5-4.5)	79	4.2 (3.1-5.2)	−0.64 (−1.3 to 0)	−0.15	.05
	12 months	75	3.1 (2.0-4.3)	72	4.1 (2.9-5.2)	−0.9 (−1.6 to −0.2)	−0.21	.007
**Depression (0-21)**								
	Baseline	100	4 (2.5-5.6)	96	4.2 (2.6-5.9)	—	—	—
	3 months	85	2.6 (1.5-3.7)	78	3.2 (2.1-4.3)	−0.61 (−1.3 to 0.1)	−0.12	.09
	12 months	75	2.4 (1.3-3.6)	72	3 (1.9-4.2)	−0.6 (−1.3 to 0.1)	−0.12	.09

^a^For symptoms, quality of life, sport and recreation, self-efficacy, active pain coping, and locus of control, a higher score indicates an improvement. For sedentary behavior, tiredness, pain, passive pain coping, anxiety and depression a lower score indicates an improvement. Results are based on GEE analyses and adjusted for corresponding baseline variables, age, OA location, and gender.

^b^I-C: difference between intervention and control group.

## Discussion

### Principal Findings

To date, unfortunately, a vast majority of patients with knee and/or hip OA remain sedentary and receive no help in the promotion of PA. Since a physically active lifestyle has been positively associated with physical function and pain [45], effective and accessible PA programs are needed. Findings from other Web-based PA interventions have been mixed [22,46,47]. With respect to the PASE questionnaire, this randomized controlled trial demonstrated that the Join2move intervention has the potential to improve PA behavior. Effect sizes for PA ranged between 0-0.19 and are congruent with findings from a meta-analysis that found an overall mean effect of 0.14 [22]. At 3 months and 12 months, PA scores in the intervention group increased with 1% (1 point) and 6% (11 points) compared to baseline. Objectively obtained PA yielded different patterns. The intervention group remained stable while the control group reported a PA reduction of 37 minutes after 3 months and 57 minutes after 1 year. A possible explanation, also highlighted in other studies [48,49] is that self-reports tend to overestimate follow-up PA levels when compared to objective monitoring by accelerometry. At the same time, accelerometer measurements are unable to register water activities such as swimming. Since swimming is a popular recreational activity for older adults in the Netherlands, underestimation of objective PA may have occurred.

Besides PA, we also found significant short-term improvements in the primary outcomes physical function and self-perceived effect. Over the long term, however, we found no significant effects for physical function and self-perceived effect. At 3 months and 12 months, physical function in the intervention group improved 15% (9 points) compared to baseline. According to a study by Roos et al [34], these values achieved the threshold of clinically meaningful improvement. Apart from the observed improvements in the primary outcome measures, we found beneficial effects for other physical (pain and fatigue) and psychological factors (self-efficacy, pain coping, and anxiety) in favor of the intervention group.

Since long-term follow-up studies demonstrated that effects of (Web-based) interventions are not sustained in the long term [21,22,50], we expected short-term rather than long-term PA effects. Surprisingly, we found only long-term effects in total PA. These results were confirmed by both self-reported and accelerometer data. Absence of short-term effects can be partly explained by improved self-reported PA outcomes in the control group. The potential presence of the so-called Hawthorne effect may have contributed to an overestimation of PA scores in the control group. Selective dropout, which may have enhanced the effects in the control group, was not found. A definitive explanation for the nonsignificant short-term differences remains unclear.

Several factors may have contributed to the success of the Join2move intervention. First, the program is the first Web-based PA intervention that focuses specifically on knee and hip OA. The intervention addresses how to perform PA despite the presence of pain. The gradual increase of activities changes the perception that physical movement is related to pain and reinforces confidence to improve PA performance [28]. This may have led to positive psychological and health outcomes. Second, the Join2move intervention seeks to align with the day-to-day activities of people. Users perform common activities (eg, walking, cycling) that are easy to integrate in their daily routine. Third, over the course of 1 year, we systematically developed and evaluated the Join2move intervention. End-users considered the intervention as user-friendly and helpful [27], which is a prerequisite for effective Web-based interventions.

Nonusage attrition has been acknowledged as a common concern in the field of Web-based education [51]. In particular, interventions, such as Join2move*,* that use automatic functions with minimal human involvement suffer from substantial rates of nonusage. In this study, 94.0% of the participants actually started the program, 46.0% reached the adherence threshold of 6 out of 9 modules, and 19.0% finished all 9 weekly modules. When considered in light of other studies, these adherence rates can be interpreted as reasonable. Previous studies by Wanner et al [52] and Connon et al [53] showed that respectively 47% and 25% of the intervention subjects never logged in to their Web-based program. Similarly, in a Web-based intervention by Hansen and colleagues [54], only 7% of the participants used the program more than once. A possible explanation for the relatively high adherence rates could be that our program incorporated automatic email reminders and website refreshments. We believe, like others [55-58], that more advanced feedback systems and regular reminders will lead to even better rates of adherence. Therefore, future research should concentrate on which strategies can improve website usage. A second factor, which may have influenced our usage rates, is the recruitment strategy used in this study. Participants were self-selected volunteers who responded to advertisements. Since self-selected participants tend to be highly educated, healthy, and already motivated to change their PA behavior, it is presumed that they have better usage rates compared to those who do not elect to participate. For example, Hansen et al [54] attributed their poor usage rates to the non-self-selected sample. This suggests that Web-based interventions, especially those without supervision, could be most suitable for those who are already willing to change their PA levels. Details about the usage and nonusage of the Join2move are described in another publication [59].

With respect to dropout attrition, 9% (4/46) adherent and 48% (26/54) nonadherent subjects did not return at least one of the follow-up surveys. This is in line with the study by Eysenbach [51], indicating that dropout and nonusage attrition are linked to each other. Since dropout rates and demographics of dropouts were similar between conditions, it is not expected that this influenced the results of the study.

As there is no cure for OA, self-management is considered a key element in the nonpharmacological treatment of knee and/or hip OA [60,61]. Self-management aims to motivate OA patients to undertake changes necessary to improve physical and psychological well-being. Although the importance is generally acknowledged, provision of self-management is underutilized. Given the clinically relevant benefits and the self-help format, Join2move could be a key component in the effort to enhance self-management in sedentary patients with knee and/or hip OA. Considering the unique potential to reach large populations through Join2move, even the small effects observed in this study could have clinical public health consequences [19]. Besides the focus on outside-care populations, patients in a care setting may also benefit from Join2move. Therefore, future work should integrate and investigate Join2move in a health care environment.

### Strengths and Limitations

First, the most important strengths are the design (ie, RCT) and the long-term character of the study. Second, we used both objective and subjective measures to assess PA. This study also has certain limitations that are important to acknowledge. First, participants were included on the basis of self-reported OA. Unfortunately, due to practical constraints, diagnosis was not confirmed through clinical tests or x-ray reports. In a previous pilot study [27], we verified self-reported OA through clinical tests. According to the American College of Rheumatology criteria [62,63], 80% had clinical knee or hip OA and 20% of the participants had no OA. These rates are in line with another validation study [64], reporting over 80% agreement between self-reported and clinically confirmed diagnoses. Although these rates are acceptable, it is presumed that we included false positive OA patients in our trial. Second, results could be biased by dropout of participants (15.6%, 31/199 at 3 months and 24.6%, 49/199 at 12 months). However, the nonresponse analysis showed similar baseline characteristics for responders, and nonresponders and dropouts were equally distributed between the intervention group and the control group. Third, with respect to the outcome variable PA, the study involved two different measures (questionnaires and accelerometers) on two occasions (3 months and 12 months). We acknowledge that this may have increased the possibility of Type I errors. Fourth, the representativeness was limited by the self-selected sample used in this study. Responders were predominantly healthy and highly educated patients. This widely recognized phenomenon is called “The inverse information law” [65]; Web-based interventions fail to reach those for whom PA behavior changes are most necessary [21,22,66-69]. In order to eliminate this issue, future studies should focus on how these specific groups could be involved in the field of Web-based education.

### Conclusions

Health care providers, such as GPs and physical therapists, may play a pivotal role in the referral of patients to Web-based interventions. Furthermore, it will be important to translate Web-based interventions, such as Join2move, to other self-help formats (eg, videos, brochures, and self-help books)**.**

## Multimedia Appendix 1

Screenshots of the Join2move intervention.

## Multimedia Appendix 2

CONSORT-EHEALTH checklist V1.6.2 [24].

## Abbreviations

BGA: behavior graded activity
BMI: body mass index
ES: effect size
GEE: generalized estimated equations
GP: general practitioner
HADS: Hospital Anxiety and Depression Scale
HOOS: hip OA outcome score
KOOS: knee OA outcome score
NIVEL: Netherlands Institute for Health Services Research
OA: osteoarthritis
OR: odds ratio
PA: physical activity
PARQ: Physical Activity Readiness Questionnaire
PASE: Physical Activity Scale for the Elderly
RCT: randomized controlled trial
