# Supplementary material for: Effectiveness of a Web-Based Physical Activity Intervention in Patients With Knee and/or Hip Osteoarthritis: Randomized Controlled Trial
Source: J Med Internet Res. 2013 Nov 22;15(11):e257. doi: 10.2196/jmir.2662 (PMC3841352; doi:10.2196/jmir.2662)

Homepage

Artrose in Beweging.nl

Persoonlijk beweegprogramma

nivel

E-mailadres ...

••••••••••••••

Inloggen

☐ Laat mij aangemeld blijven

Home | Over artrose | Leven met artrose | Programma & Onderzoek | Contact

Inschrijven voor het programma?

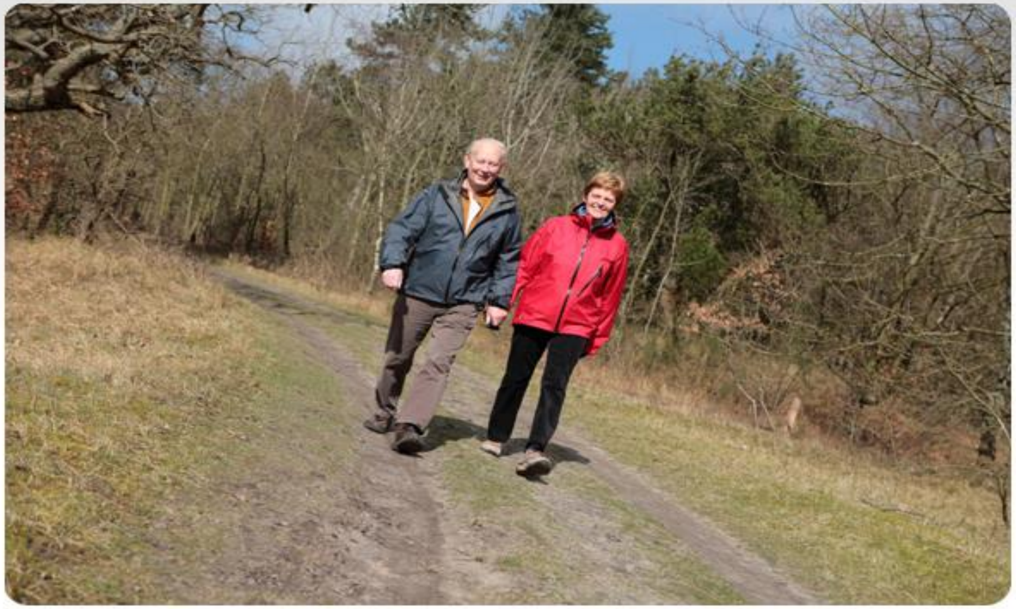

Meneer Verveer (61) - *"Artrose In Beweging was voor mij de stok achter de deur om meer te bewegen. Ik vind het heerlijk om samen met mijn vrouw te wandelen en te genieten van de natuur"*

Bewegen helpt!

Gedoseerd bewegen houdt bij mensen met artrose de conditie op peil, spieren sterk, gewrichten soepel en geest gezond. Hierdoor worden dagelijkse activiteiten, zoals wandelen, tuinieren en fietsen gemakkelijker uitvoerbaar.

Bent u tussen de 50 en 70 jaar, heeft u last van artrose in de heup en/of knie en wilt u stapsgewijs meer gaan bewegen? Schrijf u dan in voor het beweegprogramma.

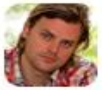

Met vriendelijke groet,
Daniël Bossen

Start onderzoek februari 2011
In februari start het NIVEL met een onderzoek naar de effectiviteit van het internet beweegprogramma. De inschrijving voor het onderzoek is verlengd tot en met juni 2011! Schrijf u hier in!

Artrose in Beweging.nl © 2010

Disclaimer - Inschrijven voor het beweegprogramma? - Contact

Stage 2 of introduction: Selection of central physical activity

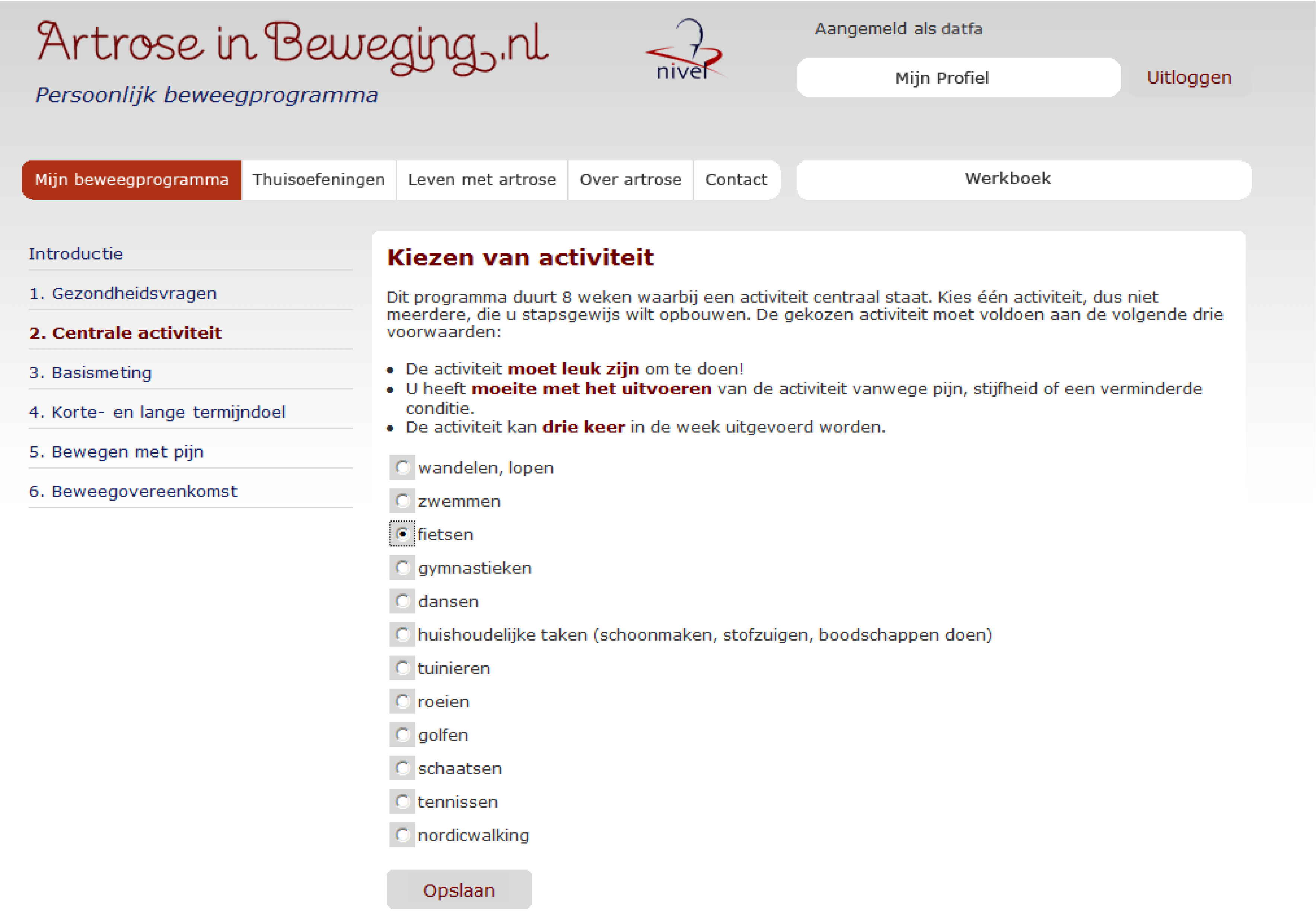

Stage 3 of introduction: A 3-day self-test

Artrose in Beweging.nl

Persoonlijk beweegprogramma

nivel

Aangemeld als datfa

Mijn Profiel

Uitloggen

Mijn beweegprogramma | Thuisoefeningen | Leven met artrose | Over artrose | Contact

Werkboek

- Introductie
- 1. Gezondheidsvragen
- 2. Centrale activiteit
- **3. Basismeting**
- 4. Korte- en lange termijndoel
- 5. Bewegen met pijn
- 6. Beweegovereenkomst

Bepalen van huidig niveau

Uw niveau van de activiteit fietsen wordt bepaald aan de hand van een driedaagse meting. Op drie verschillende dagen voert u de activiteit fietsen uit zoals u dat nu gewend bent. Hierna noteert u het aantal minuten en de pijn die u voelde tijdens de activiteit. Deze gegevens kunt u op een willekeurig moment invullen in de onderstaande tabel. Probeer de meting binnen één week af te ronden. U kunt zich nu afmelden en op een willekeurig tijdstip terugkeren.

Let op! In het verleden is gebleken dat deelnemers snel geneigd zijn zichzelf te overschatten. Vult u de gegevens zo realistisch mogelijk in.

Dag	Datum	Aantal minuten		Pijn tijdens activiteit: van 0-10 (0= geen pijn, 10= ondraaglijke pijn)
1	02-04-2013	47	min.	6
2	04-04-2013	34	min.	8
3	06-04-2013	65	min.	○ 0 ○ 1 ○ 2 ○ 3 ◉ 4 ○ 5 ○ 6 ○ 7 ○ 8 ○ 9 ○ 10

Opslaan

Stage 4 of introduction: Determining a short term goal

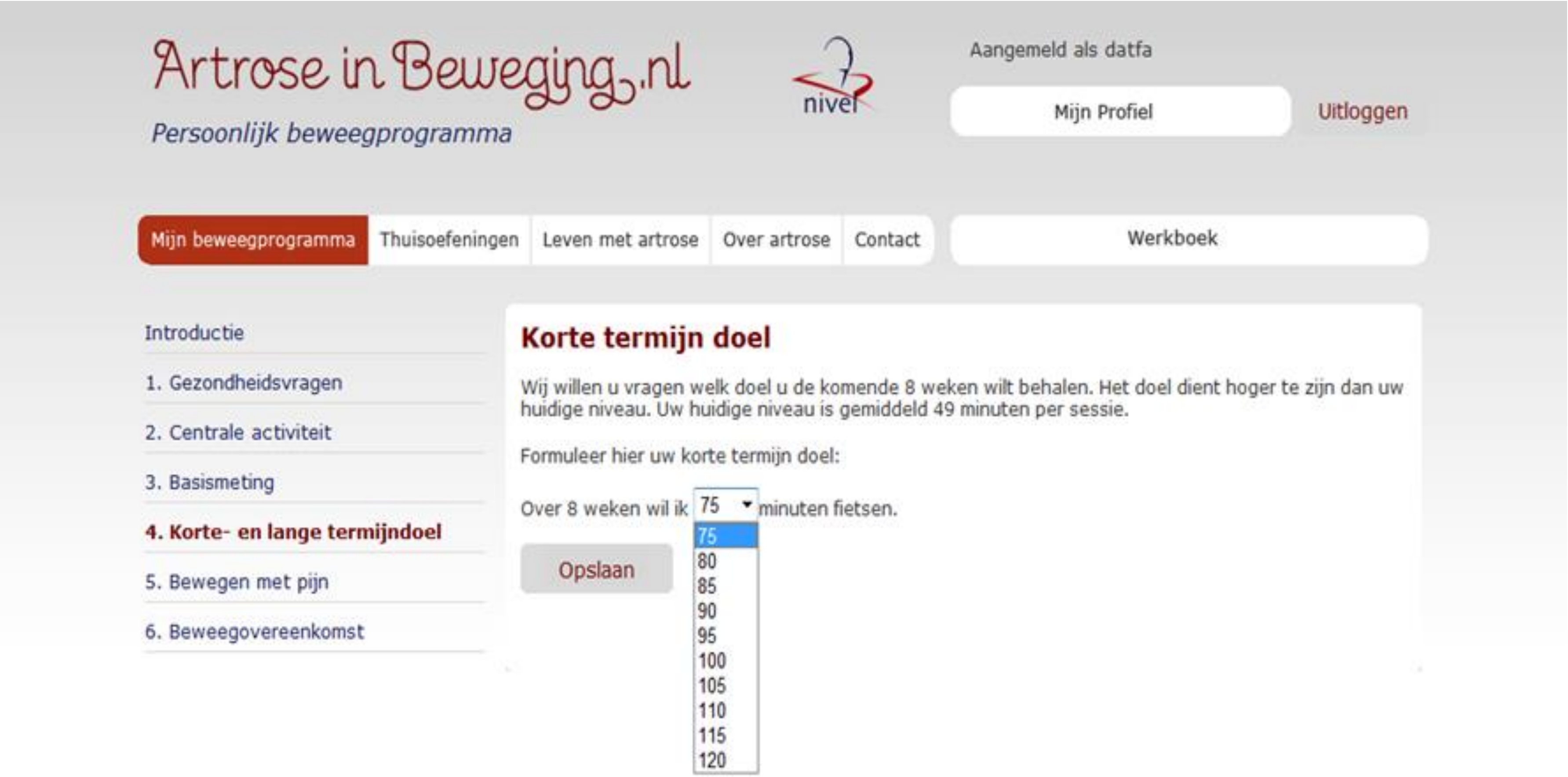

Stage 6 of introduction: Summary and agreement form

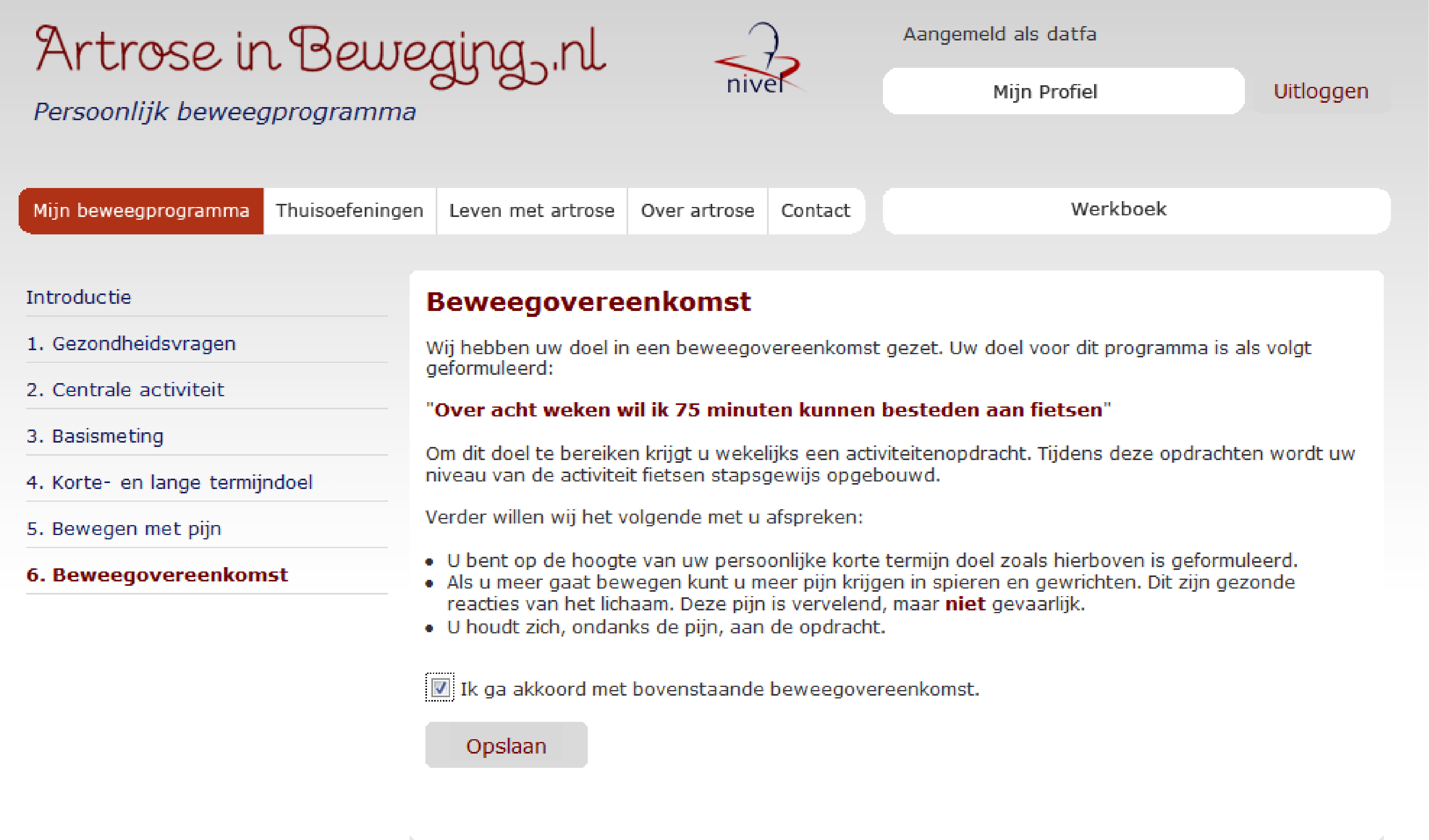

Artrose in Beweging.nl

Persoonlijk beweegprogramma

nivel

Aangemeld als datfa

Mijn Profiel

Uitloggen

Mijn beweegprogramma | Thuisoefeningen | Leven met artrose | Over artrose | Contact

Werkboek

Introductie

1. Gezondheidsvragen
2. Centrale activiteit
3. Basismeting
4. Korte- en lange termijndoel
5. Bewegen met pijn
6. **Beweegovereenkomst**

Beweegovereenkomst

Wij hebben uw doel in een beweegovereenkomst gezet. Uw doel voor dit programma is als volgt geformuleerd:

"**Over acht weken wil ik 75 minuten kunnen besteden aan fietsen**"

Om dit doel te bereiken krijgt u wekelijks een activiteitenopdracht. Tijdens deze opdrachten wordt uw niveau van de activiteit fietsen stapsgewijs opgebouwd.

Verder willen wij het volgende met u afspreken:

- U bent op de hoogte van uw persoonlijke korte termijn doel zoals hierboven is geformuleerd.
- Als u meer gaat bewegen kunt u meer pijn krijgen in spieren en gewrichten. Dit zijn gezonde reacties van het lichaam. Deze pijn is vervelend, maar **niet** gevaarlijk.
- U houdt zich, ondanks de pijn, aan de opdracht.

☑ Ik ga akkoord met bovenstaande beweegovereenkomst.

Opslaan

The first of eight week modules

Artrose in Beweging.nl

Persoonlijk beweegprogramma

nivel

Aangemeld als test 1 dafda

Mijn Profiel | Uitloggen

Mijn beweegprogramma | Thuisoefeningen | Leven met artrose | Over artrose | Contact | Werkboek

Opdracht 1
Opdracht 2
Opdracht 3
Opdracht 4
Opdracht 5
Opdracht 6
Opdracht 7
Opdracht 8
Einde

Opdracht 1

Doe op drie verschillende dagen een kleine fietstocht van ongeveer 35 minuten

Probeer, ondanks eventuele pijn, precies te doen wat er in deze opdracht staat. Doe niet minder, maar ook niet meer. Na een week ontvangt u een nieuwe opdracht op deze website. U kunt nu eventueel het werkboek invullen. U kunt zich nu afmelden en op een willekeurig moment terugkeren.

Vertrouwen

Hoeveel vertrouwen heeft u op dit moment dat de opdracht, zoals hierboven staat beschreven, gaat lukken? (0= helemaal geen vertrouwen 10= het volste vertrouwen)

○0 ○1 ○2 ○3 ○4 ○5 ○6 ○7 ○8 ◉9 ○10

Opslaan

Opbouw van uw programma

49 min.

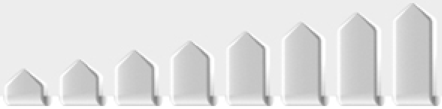

75 min.

Begin niveau | Opbouw van uw programma - 8 opdrachten | Eind doel

Evaluation form module 1

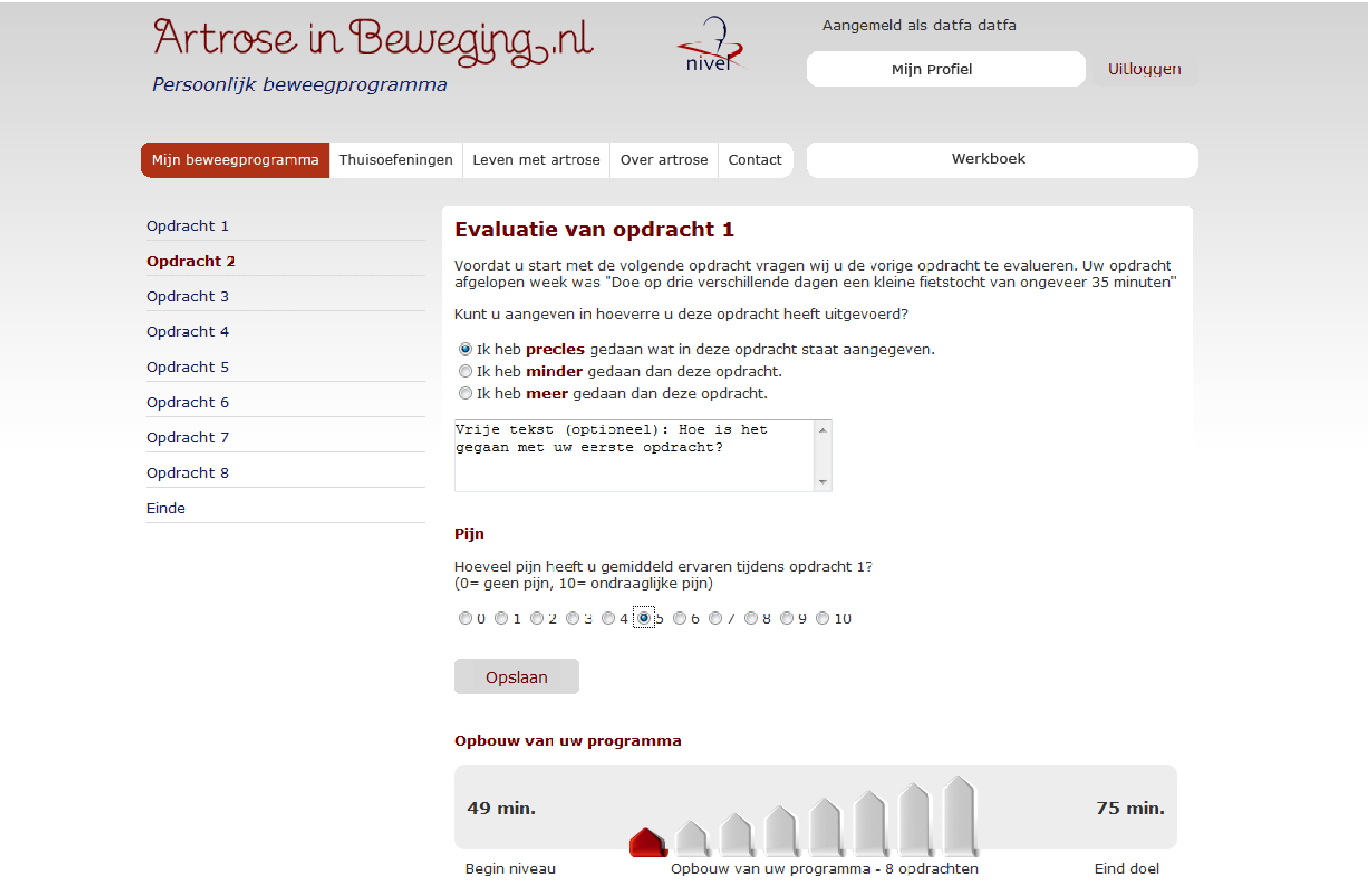

Information about OA and lifestyle

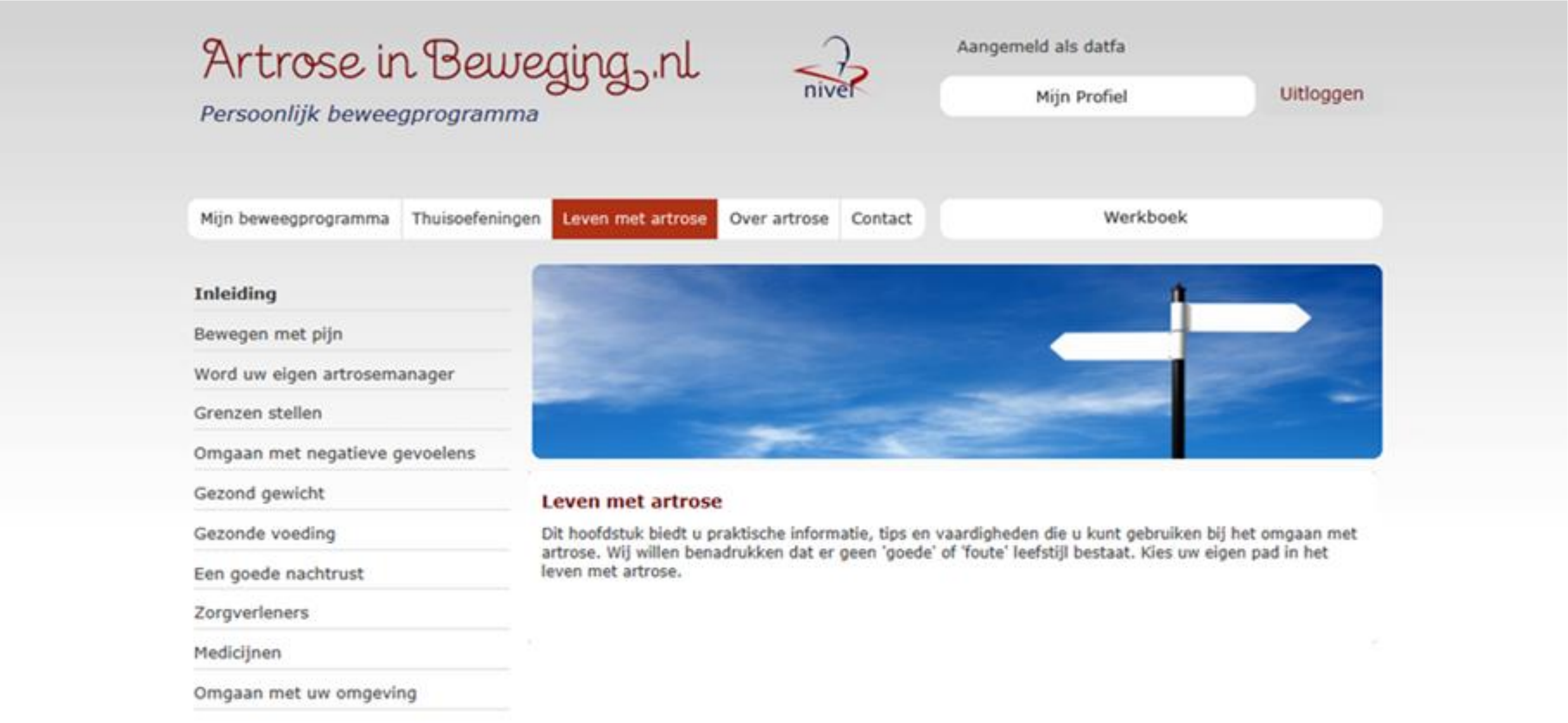

Additional strength and mobility exercises

Artrose in Beweging.nl

Persoonlijk beweegprogramma

nivel

Aangemeld als datfa

Mijn Profiel | Uitloggen

Mijn beweegprogramma | Thuisoefeningen | Leven met artrose | Over artrose | Contact | Werkboek

- Inleiding
- Spierkracht knie - oefening 1
- Spierkracht knie - oefening 2
- Spierkracht knie - oefening 3
- Spierkracht heup - oefening 4
- **Bewegelijkheid knie en heup - oefening 5**
- Spierkracht heup - oefening 6
- Spierkracht knie - oefening 7
- Spierkracht heup - oefening 8

Oefening 5: Bewegelijkheid knie en heup

Uitgangshouding:
Ga op uw rug liggen. Buig het been waar u niet mee oefent en strekt het te trainen been.

Uitvoering:
Buig uw gestrekte knie en schuif uw hiel naar uw billen toe. Zorg ervoor dat uw voeten contact blijven houden met de vloer. Wanneer u de voet zo ver mogelijk naar uw billen heeft gebracht trekt u met beide handen uw knie naar uw borst. Houd deze houding een vijftal seconden vast en breng vervolgens uw knieweer in strekpositie. Herhaal deze oefening 8-12 keer, neem even rust en doe dit dan nog twee keer. Dus drie series van 8-12 herhalingen. Herhaal dit ook met uw andere been.

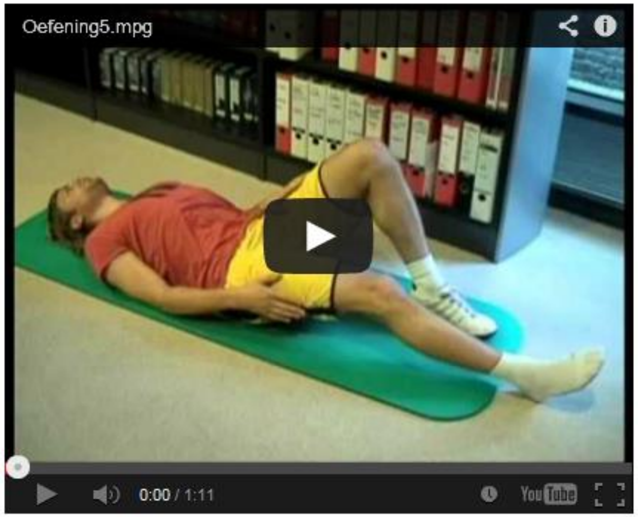

Supplement: Supplementary file 1 [file jmir_v15i11e257_app1.pdf]
