# Supplementary material for: Effectiveness of a Web-Based Physical Activity Intervention in Patients With Knee and/or Hip Osteoarthritis: Randomized Controlled Trial
Source: J Med Internet Res. 2013 Nov 22;15(11):e257. doi: 10.2196/jmir.2662 (PMC3841352; doi:10.2196/jmir.2662)
Supplement: Supplementary file 2 [file jmir_v15i11e257_app2.pdf]

CONSORT-EHEALTH Checklist V1.6.2 Report

(based on CONSORT-EHEALTH V1.6), available at [http://tinyurl.com/consort-ehealth-v1-6].

2662

Date completed

4/10/2013 5:47:09

by

Daniël Bossen

The Effectiveness of a Web-based Physical Activity Intervention in Patients with Knee and/or Hip Osteoarthritis: A Randomized Controlled Trial

TITLE

1a-i) Identify the mode of delivery in the title

"Web-based Physical Activity Intervention "

1a-ii) Non-web-based components or important co-interventions in title

This is not relevant for our article since our intervention is fully automatic

1a-iii) Primary condition or target group in the title

"in Patients with Knee and/or Hip Osteoarthritis"

ABSTRACT

1b-i) Key features/functionalities/components of the intervention and comparator in the METHODS section of the ABSTRACT

"Based on the behavioural graded activity treatment, we developed a web-based intervention to improve PA levels in patients with knee and/or hip OA, entitled Join2move. The Join2move intervention is a self-paced nine week PA program in which patients' favourite recreational activity is gradually increased in a time-contingent way."

1b-ii) Level of human involvement in the METHODS section of the ABSTRACT

yes, in the objective section of the abstract we mentioned the level of human involvement. "a fully automated web-based PA intervention"

1b-iii) Open vs. closed, web-based (self-assessment) vs. face-to-face assessments in the METHODS section of the ABSTRACT

" Volunteers were recruited via articles in newspapers and health-related websites"
"Baseline, 3 and 12 months follow-up data were collected through online questionnaires."
"In a subgroup of participants, PA was measured objectively using accelerometers."

Bliding was not possible since we compared an intervention with a waiting list control group

1b-iv) RESULTS section in abstract must contain use data

"Of the 581 interested respondents, 199 eligible participants were randomly assigned to the intervention (n=100) or waiting list control group (n=99). Response rates of questionnaires were 84.4% after 3 months and 75.4% after 12 months. In this study, 94 (94%) participants actually started the program and 46 users (46%) reached the adherence threshold of 6 out of 9 modules completed. "

1b-v) CONCLUSIONS/DISCUSSION in abstract for negative trials

"Join2move resulted in changes in the desired direction for the primary outcome measures physical function (short term) self-perceived effect (long term). Given the benefits and its self-help format, Join2move could be a component in the effort to enhance PA in sedentary patients with knee and/or hip OA."

INTRODUCTION

2a-i) Problem and the type of system/solution

"Therefore, PA as a non-pharmacological intervention has been advocated in the treatment of OA patients [9]. "
"Numerous patients lack knowledge and skills to modify their PA routines and have negative concerns (e.g. fear of pain and catastrophizing thoughts) about the impact of PA on their joints [15,16]."
"To date, there are no web-based PA interventions for patients with knee and/or hip OA. Given the advantages of internet and its unique ability to reach outside care patients with knee and/or hip OA, we developed Join2move. "

2a-ii) Scientific background, rationale: What is known about the (type of) system

"Web-based interventions are applications available through a website with the aim to enhance understanding of a health condition and to change health behavior. Particularly, web-based interventions with minimal human contact have the potential of high reach, low costs and are accessible anytime and anywhere [19]. Previous studies among general inactive populations and patients with a chronic disease (e.g. diabetes, cardiovascular diseases and chronic obstructive pulmonary disease) have produced mixed findings regarding the effectiveness of web-based PA interventions [20-22]."

METHODS

3a) CONSORT: Description of trial design (such as parallel, factorial) including allocation ratio

"This study aimed to answer the following research question: "What is the effectiveness of the Join2move intervention in patients with knee and/or hip OA on PA, physical function and self-perceived effect in comparison with a waiting list control group?""

3b) CONSORT: Important changes to methods after trial commencement (such as eligibility criteria), with reasons

NA

3b-i) Bug fixes, Downtimes, Content Changes

yes, we included the following sentence in the method section: "No content changes were made during the trial period."

4a) CONSORT: Eligibility criteria for participants

"The eligibility criteria for participants were (i) age 50-75, (ii) self-reported OA in knee and/or hip, (iii) self-reported inactivity (<30 minutes of moderate PA three five times or less per week), (iv) no face to face consults for OA with a healthcare provider, other than GP, in the last 6 months, (v) ability to access the internet weekly and (vi) and no contra-indications to exercise without supervision. "

4a-i) Computer / Internet literacy

See comment 4a

4a-ii) Open vs. closed, web-based vs. face-to-face assessments:

"Patients with self-reported knee and/or hip OA were recruited through advertisements in Dutch newspapers and online health-related websites."

4a-iii) Information giving during recruitment

"Patients with self-reported knee and/or hip OA were recruited through advertisements in Dutch newspapers and online health-related websites. The advertisements briefly explained the purpose of the project and beneficial health effects of PA.Interested individuals were referred to an open access study website and invited to complete an online eligibility questionnaire. Participants' mail addresses were used to contact participants for online follow-up questionnaires and home addresses were used for sending an information letter, informed consent form and accelerometer."

"After randomization, all participants were informed through e-mail of their group assignment"

4b) CONSORT: Settings and locations where the data were collected

"Three online questionnaires (0, 3 and 12 months) were used for data collection and a subgroup of participants received an accelerometer to measure PA"

4b-i) Report if outcomes were (self-)assessed through online questionnaires

See 4b

4b-ii) Report how institutional affiliations are displayed

Emails, the information letter and the website had the logo of the Netherlands Institute for Health Services Research (NIVEL). This was not reported in the paper since we believe it is not relevant for the results.

5) CONSORT: Describe the interventions for each group with sufficient details to allow replication, including how and when they were actually administered

5-i) Mention names, credential, affiliations of the developers, sponsors, and owners

"Over the course of one year, a team of experts of the Netherlands institute for health services research (NIVEL) developed the program."
"Further details about the development is described elsewhere [27]."

"Competing interests: The authors Daniel Bossen and Cindy Veenhof are the creators of the Join2move intervention and the NIVEL institute is owner."

5-ii) Describe the history/development process

During the development phase an iterative design methodology [26] was used to test, analyze and refine the Join2move intervention. We conducted a focus group (n=5), in home observations (n=4), a pilot study (n=20) and interviews (n=16). Furthermore, two usability methods (heuristic evaluation and a thinking aloud approach) were applied to determine the usability of the web-based program. End-users (i.e. patients with knee and/or hip OA) were involved continuously throughout the development process. The final version was used for the RCT study.

5-iii) Revisions and updating

"No content changes were made during the trial period. "

5-iv) Quality assurance methods

see 5-ii

5-v) Ensure replicability by publishing the source code, and/or providing screenshots/screen-capture video, and/or providing flowcharts of the algorithms used

We provided some screenshots of the intervention (in Dutch) in the multimedia appendix

5-vi) Digital preservation

Only the homepage and pages about OA and the program/study are accessible. The intervention itself is only accessible with a username and a password.
"Participants are initially presented with a homepage (see Multimedia Appendix 1, Figure A or http://www.webcitation.org/67SWPomxn)"

5-vii) Access

"The password-secured PA program is available 24/7 from the homepage and is provided without charge. "

5-viii) Mode of delivery, features/functionalities/components of the intervention and comparator, and the theoretical framework

"The Join2move intervention is based on a previously developed and evaluated BGA program for patients with knee and/or hip OA [23]. The BGA program incorporates a baseline test, goal setting, time-contingent PA objectives (i.e. on fixed time points) and text messages to promote PA. An essential feature of the BGA program is the positive reinforcement of gradual PA, despite the presence of pain. The gradual increase in activities changes the perception that PA is related to pain and reinforces confidence to improve PA performance [28]. This may lead to positive physical (e.g. physical capacity, muscle strength and joint mobility) and psychological changes (e.g. self-esteem, pain perception and anxiety). These health benefits are broadly comparable to the results of physiotherapy treatments."
"Participants are initially presented with a homepage (see Multimedia Appendix 1, Figure A or http://www.webcitation.org/67SWPomxn). The password-secured PA program is available 24/7 from the homepage and is provided without charge. In keeping with the BGA treatment, the Join2move intervention is a self-paced nine week PA program in which patients' favourite recreational activity is gradually increased in a time-contingent way. In the first week of the program, users select a central activity such as cycling, walking or gardening (see Multimedia Appendix 1, Figure B), perform a 3-day self-test (see Multimedia Appendix 1, Figure C) and determine a short term goal for the next eight weeks (see Multimedia Appendix 1, Figure D). Based on test performances and a short term goal, eight tailored weekly modules are automatically generated (see Multimedia Appendix 1, Figure E). Every week, new assignments and evaluation forms about pain and performance (see Multimedia Appendix 1, Figure F) are posted on the password-secured website. If a scheduled weekly module is missed, users can choose to repeat the module, adapt the difficulty or continue with the next module. In addition to the weekly PA modules, information about OA and lifestyle (see Multimedia Appendix 1, Figure G) and videos of exercises (see Multimedia Appendix 1, Figure H) are supplemented."

5-ix) Describe use parameters

"Since personal messages are updated on a weekly basis, users are encouraged to log in once a week. "

5-x) Clarify the level of human involvement

"The Join2move intervention is a fully-automated web-based intervention which contains automatic functions (automatic text messaging and automatic e-mails) without human support. "

5-xi) Report any prompts/reminders used

"Automatic e-mails are generated if participants do not visit the website regularly."
"An e-mail and telephone reminder was used when participants failed to complete their online questionnaire within two weeks. Apart from sending accelerometers and telephone reminders, the study used an automatic design. There were no face to face contacts with study subjects."

5-xii) Describe any co-interventions (incl. training/support)

NA

6a) CONSORT: Completely defined pre-specified primary and secondary outcome measures, including how and when they were assessed

"Three online questionnaires (0, 3 and 12 months) were used for data collection and a subgroup of participants received an accelerometer to measure PA. Questionnaires were created by online survey experts from the NIVEL institute and tested among a pilot study of 20 participants prior the RCT study [27]. All participants received an e-mail with an uniform resource locator link to an online questionnaire. We offered no incentives to complete questionnaires."

6a-i) Online questionnaires: describe if they were validated for online use and apply CHERRIES items to describe how the questionnaires were designed/deployed

The used questionnaires are validated as hard-copy instruments. Online versions were tested in a previous conducted pilot study among twenty participants. "Questionnaires were created by online survey experts from the NIVEL institute and tested among a pilot study of 20 participants prior the RCT study [27]."

6a-ii) Describe whether and how "use" (including intensity of use/dosage) was defined/measured/monitored

"Program usage was measured by the number of weekly modules completed. A module consisted of a text based assignment and accompanying evaluation form which was presented on the website for seven consecutive days. Once a participant read the weekly assignments and filled out the evaluation form, the module was defined as completed and the user was automatically presented with a new module. In total, there were nine week modules that could have been opened by the participant. This was automatically registered. Adequate program use was defined if users completed at least six out of nine modules."

6a-iii) Describe whether, how, and when qualitative feedback from participants was obtained

NA

6b) CONSORT: Any changes to trial outcomes after the trial commenced, with reasons

N.A

7a) CONSORT: How sample size was determined

7a-i) Describe whether and how expected attrition was taken into account when calculating the sample size

"Since no previous research has provided adequate statistical information on PA, power calculations were based on physical function and self-perceived effect. We needed 200 patients with knee and/or hip OA in total to detect a small to medium effect (0.2-0.5) in the outcome measure physical functioning and self- perceived effect (25% difference). "

7b) CONSORT: When applicable, explanation of any interim analyses and stopping guidelines

N.A

8a) CONSORT: Method used to generate the random allocation sequence

"When baseline assessments were completed, participants were randomly assigned to the intervention (n=100) or control group (n=99). "After randomization, all participants were informed through e-mail of their group assignment. Participants in the intervention group received an username and password to log in. "

8b) CONSORT: Type of randomisation; details of any restriction (such as blocking and block size)

see answer 8a above

9) CONSORT: Mechanism used to implement the random allocation sequence (such as sequentially numbered containers), describing any steps taken to conceal the sequence until interventions were assigned

"A researcher (CV), not involved in data collection, distributed sequentially numbered opaque sealed envelopes with allocation details. "

10) CONSORT: Who generated the random allocation sequence, who enrolled participants, and who assigned participants to interventions

The second author distributed sequentially numbered opaque sealed envelopes, the first author assigned participants by e-mail to the intervention or waiting list.

11a) CONSORT: Blinding - If done, who was blinded after assignment to interventions (for example, participants, care providers, those assessing outcomes) and how

11a-i) Specify who was blinded, and who wasn't

Researchers and participants were not blinded. "Due to the nature of the study (waiting list controlled), study staff and participants were not blinded to group assignment."

11a-ii) Discuss e.g., whether participants knew which intervention was the "intervention of interest" and which one was the "comparator"

see answer 11a-i above

11b) CONSORT: If relevant, description of the similarity of interventions

participants in the control group had the ability to visit the homepage of the intervention. The homepage contains information about OA, lifestyle and PA. However, controls had no access to the intervention and were not encouraged to visit the website.

12a) CONSORT: Statistical methods used to compare groups for primary and secondary outcomes

"Findings were analyzed using an intention to treat analyses. A Generalized Estimating Equations (GEE) approach controlling for baseline values, age, OA location and gender was used to analyze effects of the intervention on primary and secondary outcomes. T-tests and chi-square tests were used to compare baseline characteristics in the intervention and control group to perform non-response analysis and to determine selective attrition. Between-group effect sizes (ES) were calculated according to Cohen's d. "

12a-i) Imputation techniques to deal with attrition / missing values

"Since GEE analysis are tolerant to data missing, no imputation techniques were used [44]."

12b) CONSORT: Methods for additional analyses, such as subgroup analyses and adjusted analyses

"Complementary to the primary analysis, per protocol analyses was employed that used only adherent patients in the intervention group (at least 6 out of 9 modules completed) and the entire control group. A non-response analysis was carried out in order to examine differences among participants who completed the questionnaires and participants who did not. Furthermore, we compared primary baseline variables between the response and the non-response group in order to investigate selective attrition. "

RESULTS

13a) CONSORT: For each group, the numbers of participants who were randomly assigned, received intended treatment, and were analysed for the primary outcome

see figure 1 in manuscript

13b) CONSORT: For each group, losses and exclusions after randomisation, together with reasons

see figure 1 in manuscript

13b-i) Attrition diagram

see figure 2 in the manuscript

14a) CONSORT: Dates defining the periods of recruitment and follow-up

"Allocation ratio was 1:1 and enrollment started on January 3, 2011 and ended November 5, 2011."

14a-i) Indicate if critical "secular events" fell into the study period

No secular events occured during the study period

14b) CONSORT: Why the trial ended or was stopped (early)

N.A

15) CONSORT: A table showing baseline demographic and clinical characteristics for each group

see table 1 in the manuscript

15-i) Report demographics associated with digital divide issues

not shown

16a) CONSORT: For each group, number of participants (denominator) included in each analysis and whether the analysis was by original assigned groups

16-i) Report multiple "denominators" and provide definitions

see table 1, 2 and 3 in the manuscript

16-ii) Primary analysis should be intent-to-treat

Results of the primary intention to treat analyses are reported in table 2 and 3. We also performed secondary analyses comparing users and non-users.

17a) CONSORT: For each primary and secondary outcome, results for each group, and the estimated effect size and its precision (such as 95% confidence interval)

These results are reported in table 2 and table 3.

17a-i) Presentation of process outcomes such as metrics of use and intensity of use

"Of the 100 participants who received a password and username to enroll, 94% made a start with the first module and 6% never logged in to their personal website. Figure 2 depicts an overview of the module completion rate. The first module was completed by 80% of the subjects. This percentage declined to 55% during the second module. This percentage of completed modules remained steady up to the end of the program. Of the 94 participants who started the program, the average completion was 5.6 (SD=2.9) out of nine modules. Participants selected walking (46%), cycling (32%), Nordic walking (4%), gardening (4%) and other activities (8%) as central activity and 6% of the potential users never selected an activity. Since personal messages were updated on a weekly basis, patients had the opportunity to complete a module within seven days. When a module was missed, users still had the ability to complete the next module. Finally, 19% of the participants fulfilled all modules of the program and 46% of the subjects reached the threshold of adherence (executed at least 6 out of 9 modules)."

17b) CONSORT: For binary outcomes, presentation of both absolute and relative effect sizes is recommended

With respect to self-perceived effect (improved vs not-improved), we provided odds ratios with 95% CI as effect size and the number of participants (and%) who were improved/not-improved (see table 2)

18) CONSORT: Results of any other analyses performed, including subgroup analyses and adjusted analyses, distinguishing pre-specified from exploratory

"Those who did not complete follow-up questionnaires were more likely to have at least one comorbidity (p=<0.01) than those who did. With respect to other baseline characteristics, no differences were found (data not shown). The subgroup of participants (n=83) who wore an accelerometer did not differ from the other participants (n=116) on baseline characteristics (data not shown)."

"The per-protocol analysis, a comparison of the adherent patients in the intervention group (i.e. participants who completed 6 out of 9 week modules) and the entire control group, yielded positive self-perceived effects in favor of the intervention group (data not presented). Higher levels of participation had no influence on other primary and secondary outcomes (data not presented)."

18-i) Subgroup analysis of comparing only users

See answer 18 above

19) CONSORT: All important harms or unintended effects in each group

"Adverse events, such as extreme pain and injuries, were not reported during the intervention period "

19-i) Include privacy breaches, technical problems

NA

19-ii) Include qualitative feedback from participants or observations from staff/researchers

for this study, no qualitative data was used

DISCUSSION

20) CONSORT: Trial limitations, addressing sources of potential bias, imprecision, multiplicity of analyses

20-i) Typical limitations in ehealth trials

"Second, results could be biased by drop-out of participants (16% at 3 months and 25% at 12 months). However, the non-response analysis showed similar baseline characteristics for responders and non-responders and drop-outs were equally distributed between the intervention group and the control group. Third, the representativeness was limited by the self-selected sample used in this study. Responders were predominantly healthy and highly educated patients.

21) CONSORT: Generalisability (external validity, applicability) of the trial findings

21-i) Generalizability to other populations

"Responders were predominantly healthy and highly educated patients. This widely recognized phenomenon is called the "The inverse information law" [59]. Web-based interventions fail to reach those whom PA behavior changes are most necessary [21,22,60]. In order to eliminate this issue, future studies should focus on how these specific groups could be involved in the field of web-based education. Healthcare providers, such as GPs and physical therapists, may play a pivotal role in the referral of patients to web-based interventions. Furthermore, it will be important to translate web-based interventions, such as Join2move, for other self-help formats (e.g. videos, brochures and self-help books)."

21-ii) Discuss if there were elements in the RCT that would be different in a routine application setting

not reported

22) CONSORT: Interpretation consistent with results, balancing benefits and harms, and considering other relevant evidence

22-i) Restate study questions and summarize the answers suggested by the data, starting with primary outcomes and process outcomes (use)

yes we did, see discussion section in manuscript

22-ii) Highlight unanswered new questions, suggest future research

"A definitive explanation for the non-significant short term differences remains unclear."
"Therefore, future research should concentrate on which strategies can improve website usage."
"Therefore, future work should integrate and investigate Join2move in a health care environment. "
"In order to eliminate this issue, future studies should focus on how these specific groups could be involved in the field of web-based education. Healthcare providers, such as GPs and physical therapists, may play a pivotal role in the referral of patients to web-based interventions. Furthermore, it will be important to translate web-based interventions, such as Join2move, for other self-help formats (e.g. videos, brochures and self-help books)."

Other information

23) CONSORT: Registration number and name of trial registry

The Netherlands National Trial Register. Trial number: NTR2483

24) CONSORT: Where the full trial protocol can be accessed, if available

No. Only avalabile in Dutch language

25) CONSORT: Sources of funding and other support (such as supply of drugs), role of funders

This study was funded by a non governmental foundation.

X26-i) Comment on ethics committee approval

"Ethics approval was obtained from the medical ethics committee of the VU University Medical Center Amsterdam. " Trial number: NTR2483

x26-ii) Outline informed consent procedures

Informed consent was obtained offline by post. The attached letter contained information about the web-based intervention, practical information about the study and potential benefits, disadvantages and risks to participate in the study. Moreover, we provided an description how we ensure the confidentiality of personal data and how we respect the rights of privacy.

"Interested patients who met the inclusion criteria were sent an invitation letter with informed consent. Once written informed consent was obtained, participants were invited to fill out an online baseline questionnaire."

X26-iii) Safety and security procedures

"One of the inclusion criteria was no contra-indications to exercise without supervision. "This last criteria was determined by the PA readiness questionnaire (PARQ) [25]. The PARQ questionnaire is designed to identify persons for who increased PA may be contraindicated. If participants answered “yes” to any of the seven PARQ, they were advised to see their GP before participation. If patients filled out ‘no’ to all of the questions, it was considered safe for the patients to engage Join2move."

X27-i) State the relation of the study team towards the system being evaluated

No financial or other conflicts of interest are declared. The authorsare also the creators of the Join2move intervention.
